# Characteristics and immunoprotective functions of three cysteine proteases from *Clonorchis sinensis*

**DOI:** 10.3389/fimmu.2025.1550775

**Published:** 2025-04-03

**Authors:** Yunliang Shi, Xiaoqin Li, Kai Liang, Ting Lu, Yu Chen, Yashi Lai, Yaoting Li, Shuai Wei, Shanshan He, Lili Tang, Dengyu Liu, Yanwen Li

**Affiliations:** ^1^ Parasitology Department, School of Basic Medical Sciences, Guangxi Medical University, Nanning, China; ^2^ Key Laboratory of Basic Research on Regional Diseases (Guangxi Medical University), Education Department of Guangxi Zhuang Autonomous Region, Nanning, China; ^3^ Department of Medical Laboratory, Shenzhen Longgang District Eighth People’s Hospital, Shenzhen, China; ^4^ Gastroenterology Department, Guangxi Zhuang Autonomous Region People’s Hospital, Nanning, China; ^5^ Department of Schistosomiasis Prevention and Control, Disease Prevention and Control Center of Hengzhou City, Hengzhou, China; ^6^ Department of Medical Laboratory, Hechi People’s Hospital, Hechi, China

**Keywords:** *Clonorchis sinensis*, cysteine proteases, characteristics, liver damage, immune protection

## Abstract

**Introduction:**

Cysteine proteases from *Clonorchis sinensis*, including various proteins, are essential for its pathogenicity and serve as potential vaccine candidates. This study assesses the protective effects of three *C. sinensis* cysteine proteases (CsCP1-3).

**Methods:**

Mice immunized with recombinant CsCP1-3 and adjuvants were subsequently infected with *C. sinensis* metacercariae after three immunization rounds. Liver damage was evaluated through hematoxylin and eosin (H&E), Masson’s trichrome, and immunohistochemical analyses. The levels of IgG1, IgG2a antibodies, and cytokines (IFN-g, IL-2, IL-4, and IL-10) were quantified by enzyme-linked immunosorbent assay (ELISA).

**Results:**

RT-qPCR revealed that CsCP1-2 exhibited the highest expression in newly encysted larvae (NEL), while CsCP3 was predominantly expressed in adult stages. Immunohistochemical localization confirmed that CsCP1-3 are present in the eggshells, syncytial layers of metacercariae, NEL cuticle, and adult intestines. Histological and immunohistochemical analysis demonstrated that the rCsCP1-3-immunized group displayed reduced liver inflammation and biliary fibrosis compared to the control group. The rCsCP1-3 induced a progressive increase in specific IgG1 and IgG2a antibody titers by the second week post-immunization. In the CsCP1-2 group, cytokines IFN-g, IL-2, IL-4, and IL-10 were elevated relative to the control, with particularly high levels of IFN-g and IL-10 in CsCP1, indicating a strong mixed Th1/Th2 immune response. In contrast, the CsCP3 immunization group exhibited a transient increase in cytokines (IFN-g, IL-2, IL-4, and IL-10) three days postinfection, which subsided after one to two weeks.

**Discussion:**

These findings suggest that CsCP1-3 elicit robust antibody and cellular immune responses, mitigating liver damage caused by *C. sinensis* infection. CsCP1, in particular, induces a potent mixed Th1/Th2 response, positioning it as a promising vaccine candidate.

## 1 Introduction

Clonorchiasis is a serious foodborne parasitic disease that affects over 200 million people worldwide, with an estimated 15-20 million individuals infected (1, 2). Infection with *Clonorchis sinensis* (*C. sinensis*) primarily occurs through the accidental ingestion of metacercariae, potentially leading to bile duct dilation, cholecystitis, cholelithiasis, liver fibrosis, and even hepatocellular carcinoma and cholangiocarcinoma (3–5). The disease is mainly endemic in southeastern and northeastern China, northern Korea, northern Vietnam, and eastern Russia, regions where the consumption of raw fish is common, complicating prevention and control efforts (6, 7). Current prevention and treatment strategies primarily rely on medications such as praziquantel and albendazole, with no commercially available vaccine yet (8, 9).

Cysteine proteases (CPs) are eosinophilic proteolytic enzymes with hydrolytic activity. They are classified into various families based on primary amino acid sequence and secondary/tertiary structure (10). In parasitic organisms, CPs are secreted proteins involved in key biological and pathogenic processes, including host cell adhesion, tissue invasion, cytotoxicity, nutrient uptake, and immune evasion (11, 12). CPs from various parasites have shown the potential to induce robust humoral and cell-mediated immune responses, positioning them as promising vaccine candidates (13). CPs derived from *Fasciola hepatica* (14), *Schistosoma mansoni* (15, 16), *Haemonchus contortus* (17), and *Trichinella* sp*iralis* (18) have been demonstrated to stimulate strong immune responses, with immunization reducing worm burden (18, 19). As such, parasitic CPs are considered significant vaccine candidates with broad potential for application (20, 21).

Several cysteine proteases have been identified in *C. sinensis*, including cathepsins B, F, L, and legumain (22–25). However, limited immunoprotection studies on CsCPs have been conducted. Notably, a 37.6 kDa CsCP has been shown to induce both humoral and cellular immune responses, providing substantial protection in Sprague-Dawley rats (26). Additionally, a 35 kDa recombinant CsCP (B.s-CotC-CsCP) has demonstrated the ability to induce a specific immune response in mice, resulting in a significant reduction in liver fibrosis post-immunization (27). Furthermore, immunization with CotC-CsCP in grass carp has proven effective in conferring resistance to *C. sinensis* infection (28). Despite these findings, the immunoprotective roles of many CsCPs remain inadequately understood.

This study examines the expression and localization of three *C. sinensis* cysteine proteases (CsCP1-3). Mice were immunized with recombinant forms of these proteases and subsequently challenged with *C. sinensis* infection to assess liver damage and measure antibody and cytokine levels. This investigation into the immunoprotective roles of CsCP1-3 aims to enhance understanding of their potential as vaccine candidates.

## 2 Materials and methods

### 2.1 *C. sinensis* metacercariae, newly excysted juvenile worms, adult worms and eggs preparation

*C. sinensis*-infected *Pseudorasbora parva* was collected from Hengzhou City, Guangxi. After the head, scales, and viscera were removed, the fish flesh was minced and digested at 37°C for 12 hours in an artificial digestive fluid (1% hydrochloric acid and 0.6% pepsin). Metacercariae were isolated under a stereomicroscope and stored in 0.9% saline solution (29). A portion of the metacercariae was treated with trypsin digestive fluid (0.025% trypsin, pH 7.4) and incubated at 37°C for 3 minutes to obtain newly excysted juveniles (NEJ). Additionally, a portion of the metacercariae was orally administered to Sprague-Dawley (SD) rats, with 150 metacercariae per rat. After 4 weeks, the rats were euthanized, and adult worms were collected from the bile ducts, along with eggs from the uteri of the adult worms. The adult worms, NEJ, metacercariae, and eggs were partially stored in RNA preservation solution at -80°C and partially fixed in 4% paraformaldehyde at room temperature.

### 2.2 Expression, purification, and identification of recombinant CsCP1-3 protein

Total RNA was extracted from adult *C. sinensis*, and cDNA was synthesized through reverse transcription. The CsCP1-3 gene (GenBank Accession: DQ902582.1, DQ902583.1, DQ902586.1) was amplified by PCR and subcloned into the pPic9k (+) expression vector(Sangon Biotech, Shanghai, China), creating the recombinant plasmid pPic9k (+)-CsCP1-3. After sequence verification, the construct was transformed into the *Pichia pastoris* GS115 strain(Sangon Biotech, Shanghai, China). GS115 cells harboring pPic9k (+)-CsCP1-3 were inoculated into 5 mL of YPD liquid medium and cultured at 30°C with shaking at 250 rpm for 12 hours. The culture was then diluted 1:50 into BMGY medium and further incubated at 30°C with shaking at 250 rpm for 12-16 hours. Following centrifugation at room temperature, the cells were resuspended in an equal volume of BMMY medium and cultured at 30°C with shaking at 250 rpm. Methanol was added every 12 hours to maintain a final concentration of 0.5%, and after 72 hours, the supernatant was collected by centrifugation at 4°C and analyzed by 12% SDS-PAGE and Western blotting. The expressed protein was purified using a Ni-agarose affinity column with a His tag and dialyzed to obtain a large quantity of the protein.

### 2.3 Preparation of anti-rCsCP1-3 immune sera

To generate anti-sera for subsequent experiments, 150 μg of rCsCP1-3 was mixed with an equal volume of complete Freund’s adjuvant(Sigma-Aidrich, USA) and administered to rats *via* multiple subcutaneous and intradermal injections. The dose was halved for the second and third booster immunizations, which were given at 2-week intervals. Blood was collected from the rats’ tails before each vaccination and 2 weeks after the final immunization (30). The serum was separated, inactivated by heating at 56°C for 30 minutes to deactivate the complement, and stored at -20°C for future use. The serum was initially diluted at 1:800 and further serially diluted, with the titer of anti-rCsCP1-3 serum determined using an ELISA method.

### 2.4 CsCP1-3 expression at different developmental stages of *C. sinensis*

Total RNA was extracted from the eggs, metacercariae, NEJ, and adult worms of *C. sinensis* using the Trizol method. cDNA was synthesized using the PrimeScript RT Master Mix kit (Takara, Japan, RR036A). Primers for CsCP1-3 were designed according to the information in **Supplementary Table 1**, with PCR amplification conditions specified therein. RT-qPCR was performed using the Takara RT-qPCR premix (RR820A, Japan), with β-Actin as the internal reference gene. Relative mRNA levels of CsCP1-3 at different developmental stages of the liver fluke were determined using the comparative cycle threshold (2−ΔΔCT) method (31).

### 2.5 Localization of CsCP1-3 in different developmental stages of *C. sinensis*

Eggs, metacercariae, NEJ, and adult worms of *C. sinensis* were fixed in 4% paraformaldehyde, paraffin-embedded, and sectioned to 4 μm thickness. The sections underwent deparaffinization using xylene and graded ethanol, followed by antigen retrieval in Sodium Citrate Antigen Retrieval Solution (pH 6.0) using an autoclave. Endogenous peroxidase activity was quenched with 3% H_2_O_2_ for 15 minutes, followed by incubation with 5% goat serum (1:10 dilution) at 37°C for 30 minutes. Subsequently, the sections were incubated overnight at 4°C in the dark with SD rat anti-CsCP1-3 serum (1:800 dilution). The following day, after equilibration to room temperature, the sections were washed four times with PBS, then incubated with Goat Anti-Rat IgG H&L (Alexa Fluor^®^ 594) (Abcam, United Kingdom) at 37°C for 1 hour with intermittent shaking. After three PBS washes, protein localization was observed using a fluorescence microscope (Leica DMi8, Germany).

### 2.6 Immunization protocol with rCsCP1-3 protein

Eight-week-old BALB/c mice were randomly assigned to five groups, each comprising nine mice. The initial immunization involved administration of 75 μg of rCsCP protein (dissolved in 50 μL) mixed with 50 μL of complete Freund’s adjuvant. For the second and third booster immunizations, incomplete Freund’s adjuvant (Sigma-Aldrich, USA) was used, with the dosage reduced by half. The primary, secondary, and tertiary immunizations were administered at two-week intervals. Control groups received PBS or PBS plus Freund’s adjuvant. Two weeks after the final immunization, each mouse was orally administered 30 metacercariae. At 0, 2, and 4 weeks post-gavage, three mice from each group were euthanized, and serum, spleen, and liver tissues were harvested for liver function assays, splenocyte culture, and histopathological analysis.

### 2.7 Histopathological assessment of liver tissue

For dehydration, liver tissues were fixed in 4% paraformaldehyde for 24 hours, followed by a graded ethanol dehydration series (70%, 80%, 90%, 95%, and 100%). After clearing with xylene, the tissues were embedded in paraffin, sectioned, and mounted. Sections were stained with hematoxylin and eosin (HE) for routine histological evaluation and Masson’s trichrome for collagen visualization. Pathological alterations were examined and documented using a light microscope (Olympus BX43, Japan). Liver inflammation was quantified using the mHAI scoring system based on HE staining (32). Fibrotic areas were morphologically evaluated and scored using the Ishak scoring system with ImageJ software after Masson’s trichrome staining (33).

For immunohistochemical analysis, antigen retrieval of mounted sections was achieved by incubation in citrate buffer (pH 6.0) for 3 minutes, followed by endogenous peroxidase blocking at room temperature for 10 minutes. After a 30-minute blocking step with 5% goat serum at 37°C, sections were incubated overnight at 4°C with primary antibodies: Anti-α-SMA Mouse mAb (1:1000 dilution) and Anti-Collagen I Rabbit pAb (1:1000 dilution) (both from Servicebio, Wuhan, China). Following washes, sections were incubated with HRP-conjugated secondary antibodies—Goat Anti-Mouse IgG (catalog number D110087-0100) and Goat Anti-Rabbit IgG (1:2000 dilution, catalog number D110058-0100) (both from Sangon Biotech, Shanghai, China)—for 45 minutes at 37°C. Immune complexes were visualized using 3,3’-diaminobenzidine (DAB), with subsequent hematoxylin counterstaining for 5 minutes and bluing. Dehydration, clearing, and microscopic examination were performed, and positive staining areas were quantified using ImageJ software. Serum levels of alanine aminotransferase (ALT) and aspartate aminotransferase (AST) in mice were determined using commercial assay kits (Nanjing Jiancheng, China, C009-2-1 for ALT and C010-2-1 for AST) according to the manufacturer’s protocol.

### 2.8 Determination of serum specific IgG, IgG1, and IgG2a antibody titers

The rCsCP1-3 antigen was coated at 50 μg per well and incubated overnight at 4°C in a humid chamber. Non-specific binding was blocked with 5% bovine serum albumin (BSA) at 37°C for 2 hours. Primary antibodies from mouse serum were added starting at a 1:200 dilution, followed by serial dilutions up to 1:51200, and incubated overnight at 4°C. After three washes with PBS-T (pH 7.4), HRP-conjugated goat anti-mouse IgG secondary antibodies (diluted 1:50000, 100 μL per well) were added (Sangon Biotech, Shanghai, China, China, catalog number D110087-0100) and incubated for 1 hour at 37°C. The plates were washed three times with PBST (pH 7.4), and the colorimetric substrate 3,3’,5,5’-tetramethylbenzidine (TMB) (100 μL per well) was added for color development. The reaction was allowed to proceed for 10-15 minutes at room temperature in the dark, then stopped by adding 1M sulfuric acid (H_2_SO_4_) (100 μL per well). Absorbance at 450 nm was measured using a microplate reader (BioTek Synergy H1). The endpoint titer for specific IgG antibodies in infected serum was determined as the highest dilution at which the measured values approximated those of the negative control serum.

### 2.9 Splenocyte preparation and cytokine measurement

The effect of rCsCP1-3 on splenocyte viability was evaluated using the CCK-8 assay (APExBIO, USA, K1018). Splenocytes were isolated and seeded into 96-well plates at a density of 5 × 10^5^ cells per well in a complete RPMI-1640 medium (containing 10% fetal bovine serum and 1% penicillin/streptomycin) (34). After 24 hours of incubation at 37°C in a 5% CO2 atmosphere, cells were treated with rCsCP1-3 at concentrations of 0, 12.5, 25, 50, 100, and 200 μg/mL (10 μL per well) for an additional 24 hours. The old medium was removed, and 10 μL of CCK-8 solution was added to each well, followed by incubation for 2 hours at 37°C in a 5% CO2 incubator. Cell viability was assessed by measuring absorbance at 450 nm using a microplate reader (BioTek Synergy H1), with cell viability expressed as a percentage. The optimal concentration for co-culture with splenocytes was determined to be 12.5 μg/mL (100 μL per well).

For cytokine measurement, splenocytes were seeded into 24-well plates at a density of 5 × 10^5^ cells/mL (1 mL per well) and treated with rCsCP1-3 at a concentration of 12.5 μg/mL (100 μL per well), with PBS serving as the negative control. After 48 hours of incubation at 37°C in a 5% CO2 environment, cell supernatants were collected and stored at -80°C for subsequent analysis. The concentrations of interleukin (IL)-2 (Multi Sciences, China, EK202), IL-4 (Multi Sciences, China, EK204), IL-10 (Multi Sciences, China, EK210), and interferon (IFN)-γ (Multi Sciences, China, EK280) in the supernatants were quantified using ELISA kits according to the manufacturer’s protocol. A multifunctional microplate reader (BioTek Synergy H1) was used to measure dual-wavelength absorbance at 450 nm and 570 nm/630 nm, with the adjusted OD values calculated by subtracting the 570 nm/630 nm readings from the 450 nm readings. The experiment was performed in triplicate, and mean values were used for analysis.

### 2.10 Statistical analysis

All quantitative data are presented as mean ± standard deviation (x ± s) from three independent experiments. Data were analyzed using one-way analysis of variance (ANOVA), followed by Bonferroni’s *post hoc* test. Statistical charts were generated using GraphPad Prism software (version 9.0; GraphPad Software, Inc.), and statistical analyses were conducted with SPSS software (version 25.0; SPSS Inc.).

## 3 Results

### 3.1 Sequence comparison and analysis

**Figure 1:**
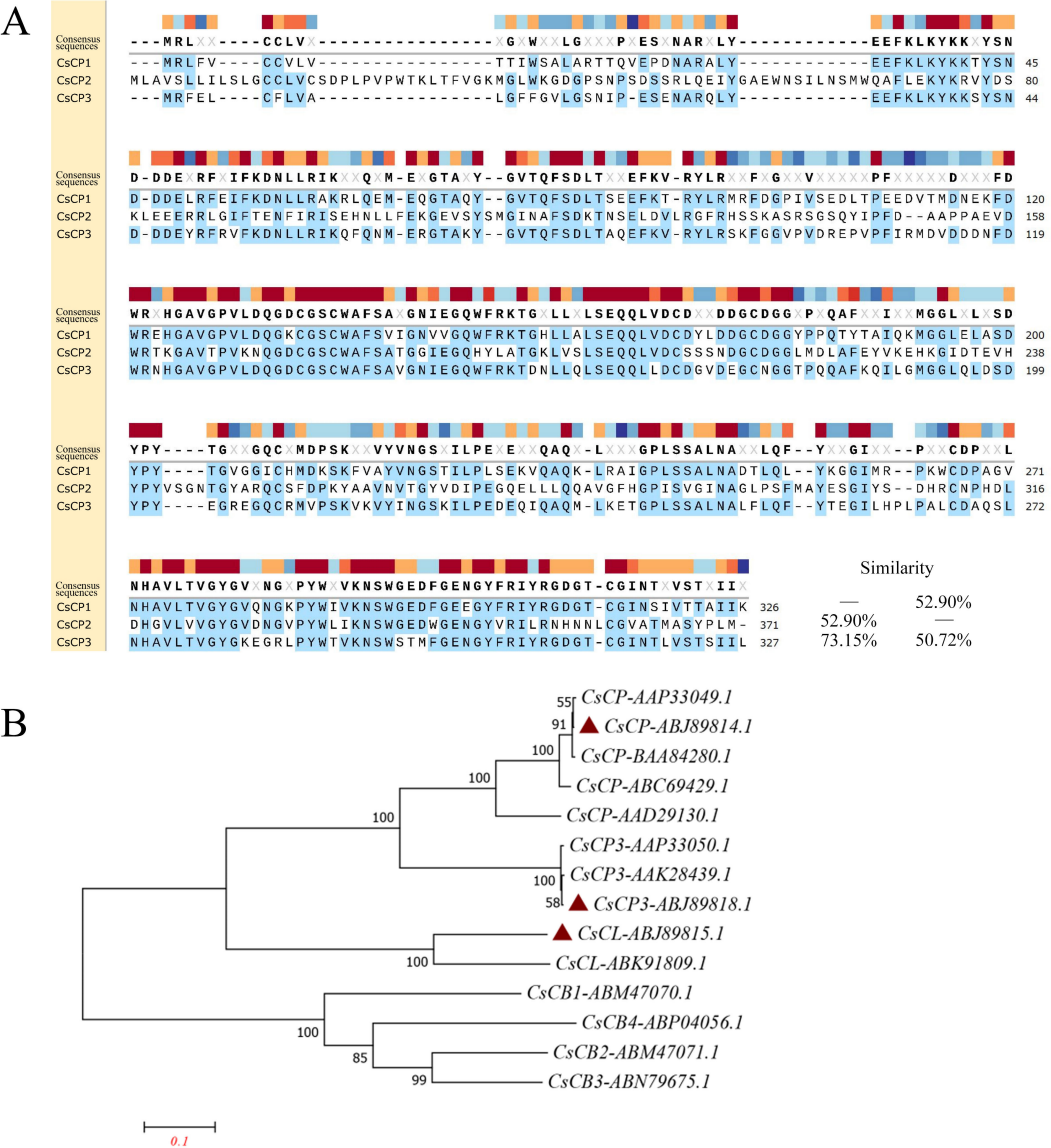
Homology and phylogenetic analysis of rCsCP1-3. **(A)** Homology analysis of the amino acid sequences of rCsCP1-3 revealed that all three proteins contain the conserved cysteine protease sequences ERFNIN, GNFD, and GCNGG, along with the catalytic residues C, H, and N. **(B)** Phylogenetic analysis of the amino acid sequences of rCsCP1-3 shows their clustering into distinct clades, with rCsCP1 and rCsCP3 being closely related. rCsCP1: ABJ89814.1; rCsCP2: ABJ89815.1; rCsCP3: ABJ89818.1.

CsCP1-3 possess conserved structural domains characteristic of cysteine proteases, including ERFNIN, GNFD, GCNGG, and the catalytic triad C, H, N (**Figure 1A**). The open reading frame (ORF) amino acid sequences of CsCP1-3 are 326, 371, and 327 amino acids in length, respectively. The amino acid sequence homology between CsCP1 and CsCP2 is 52.9%, between CsCP1 and CsCP3 is 73.15%, and between CsCP2 and CsCP3 is 50.72%. Phylogenetic analysis of *C. sinensis* cysteine proteases reveals that CsCP1, CsCP2, and CsCP3 are situated on distinct branches (**Figure 1B**). B-cell epitope prediction, performed using the IEDB tool (https://www.iedb.org/), identified multiple phosphorylation sites and B-cell epitopes across CsCP1-3, suggesting strong antigenicity and immunogenicity, making CsCP1-3 a promising candidate for vaccine development (**Supplementary Figure 1**).

### 3.2 Expression of rCsCP1-3

**Figure 2:**
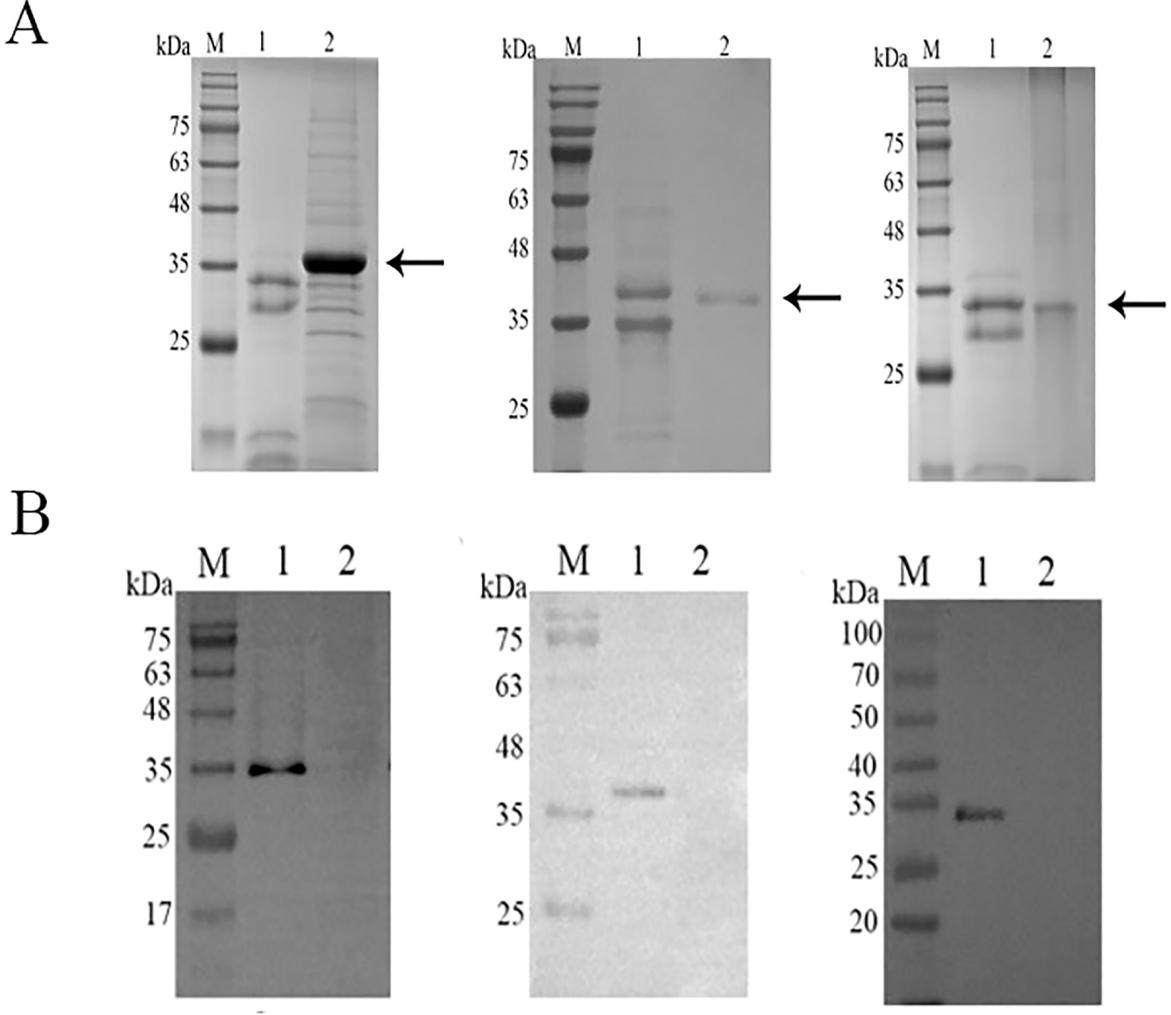
Expression and validation of rCsCP1-3 in yeast. **(A)** SDS-PAGE analysis of rCsCP1-3 purified using a nickel column (Ni-NTA), showing molecular weights of 35 kDa (rCsCP1 and rCsCP3) and 40 kDa (rCsCP2). **(B)** Western blotting of the 6 × His tag on rCsCP1-3. **(A, B)** show, from left to right, rCsCP1, rCsCP2, and rCsCP3; M, molecular weight markers; lane 1, induced protein expression of rCsCP1-3; lane 2, negative control.

The cDNA of CsCP1-3 from *C. sinensis* was cloned into the pPic9k expression vector. The recombinant proteins, expressed in *Pichia pastoris* GS115, were present in the soluble fraction. The soluble proteins were denatured with guanidine hydrochloride and purified using Ni2+-affinity chromatography (**Figure 2A**). The recombinant CsCP1-3 proteins were efficiently eluted at molecular weights of 35 kDa, 40 kDa, and 35 kDa, respectively. Western blot analysis confirmed that the target bands in the 72-hour induced supernatant were recognized by anti-6 × His monoclonal antibody (**Figure 2B**).

### 3.3 Transcriptional levels of CsCP1-3 at the egg, metacercaria, newly excysted juvenile, and adult worm stages

**Figure 3:**
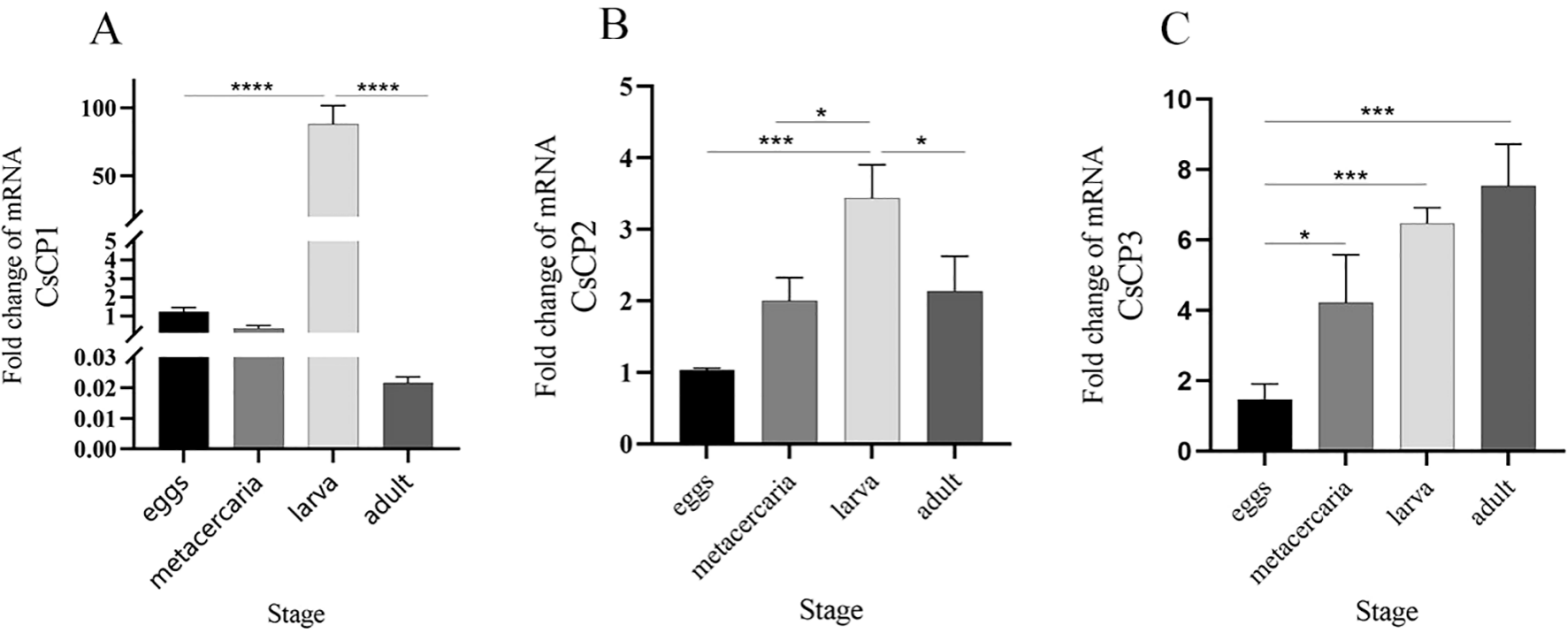
Gene expression of rCsCP1-3 at different developmental stages of *Clonorchis sinensis*. RT-qPCR was used to quantify the mRNA expression levels of rCsCP1-3 in the egg, metacercaria, newly excysted juvenile, and adult stages of *C*. *sinensis*. **(A)** rCsCP1; **(B)** rCsCP2; **(C)** rCsCP3. *P < 0.05, **P < 0.01, ***P < 0.001, ****P < 0.0001.

RT-qPCR results demonstrated that the expression of CsCP1 was significantly upregulated at the NEJ stage compared to the egg, metacercaria, and adult stages (*P* < 0.001), with expression in the NEJ stage being nearly 100-fold higher than in the other stages (**Figure 3A**). CsCP2 also exhibited the highest expression in the NEJ stage, being twice as high as in the adult and four times as high as in the egg stage (**Figure 3B**). In contrast, CsCP3 expression progressively increased across all stages, peaking in the adult worm stage (**Figure 3C**). These results suggest that CsCP1-3 have distinct roles throughout the developmental stages of *C. sinensis*.

### 3.4 Tissue localization of CsCP1-3 at different developmental stages of *C. sinensis*

**Figure 4:**
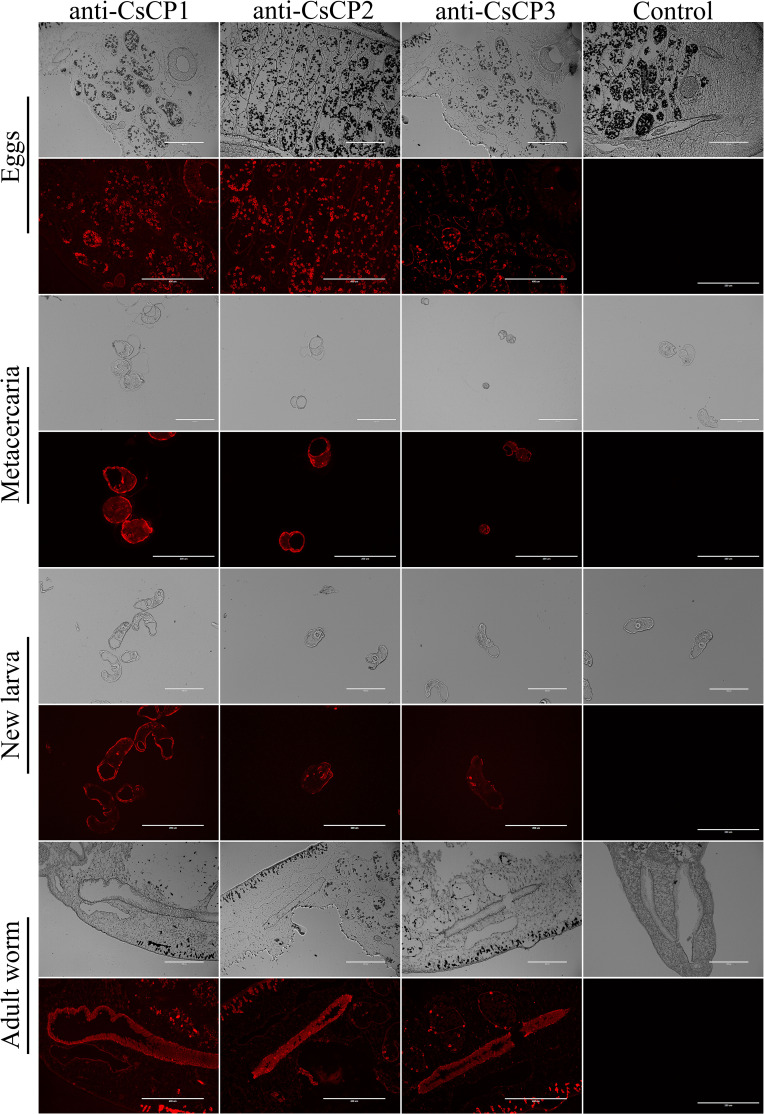
Tissue localization of rCsCP1-3 at various developmental stages of *Clonorchis sinensis*. Immunofluorescence localization of rCsCP1-3 distribution in eggs, metacercariae, newly excysted juveniles, and adult stages of *Clonorchis sinensis*.

Immunofluorescence localization revealed that CsCP1 is primarily localized on the surface of the eggshell, the surface of metacercariae, the tegument and oral sucker of NEJ, and the intestine, tegument, and cell layer of adult worms (**Figure 4**). CsCP2 and CsCP3 are predominantly found on the eggshell and metacercariae surfaces, the tegument of NEJ, and the intestine, body surface tegument, and cell layers of adult worms (**Figure 4**). No fluorescence signal was detected in the PBS-immunized rat serum, which served as a negative control (**Figure 4**).

### 3.5 Examination of liver tissue sections stained

**Figure 5:**
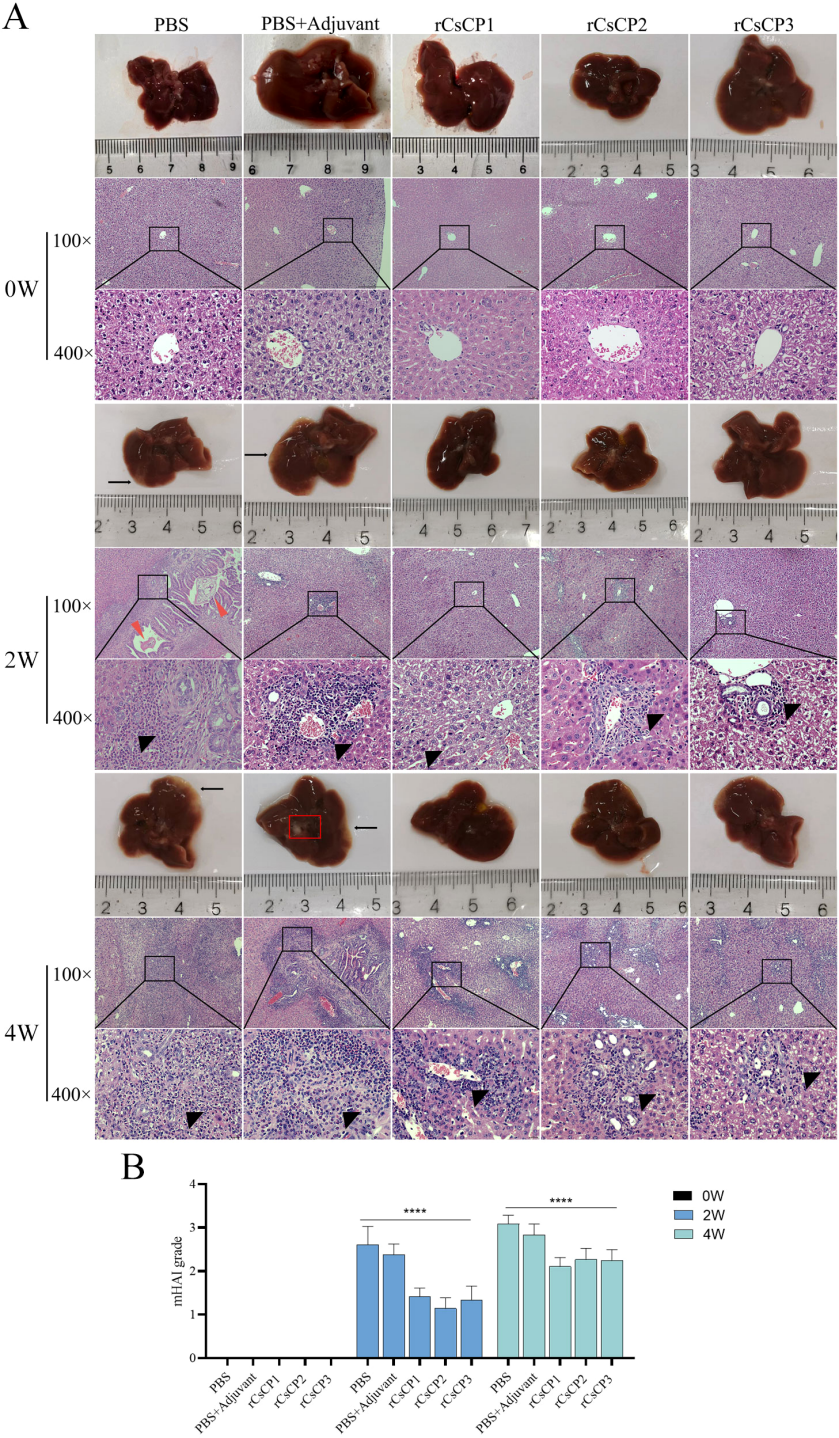
rCsCP1-3 immunization ameliorates liver damage and inflammatory infiltration induced by *Clonorchis sinensis* infection in mice. Mice immunized with rCsCP1-3 exhibited no significant pathological changes in liver morphology. In contrast, liver samples from PBS and PBS plus Freund's adjuvant-treated groups showed increased lesion sites and the formation of small cysts **(A)**. H&E staining revealed dense inflammatory cell infiltration around bile duct tissues in the PBS and PBS plus Freund's adjuvant groups compared to the rCsCP1-3 immunized group **(A)**. The degree of liver pathology in each group was assessed using the mHAI scoring system **(B)**. Note: Black arrows indicate small cysts; black triangles indicate inflammatory infiltration; red triangles indicate *C.s sinensis* juvenile worms; ****P < 0.0001. Scale bar: 100 µm.

Infection experiments demonstrated that mice immunized with rCsCP1-3, as well as those in the PBS and PBS plus Freund’s adjuvant groups, exhibited mild liver lesions one month after being challenged with *C. sinensis* metacercariae, with no significant gross appearance differences. However, H&E staining revealed a substantial influx of neutrophils and lymphocytes from the bile ducts to the surrounding tissues in the PBS and PBS plus Freund’s adjuvant groups (**Figure 5A**). In contrast, in mice vaccinated with rCsCP1-3, the inflammatory cell infiltration around the bile ducts was less pronounced (**Figure 5A**), indicating a milder degree of liver inflammation. Moreover, mHAI scores for the PBS and PBS plus Freund’s adjuvant groups were significantly higher than those for the rCsCP1-3 immunized group at both the second and fourth weeks post-infection (*P* < 0.001) (**Figure 5B**), suggesting that rCsCP1-3 immunization resulted in reduced hepatic inflammation.

**Figure 6:**
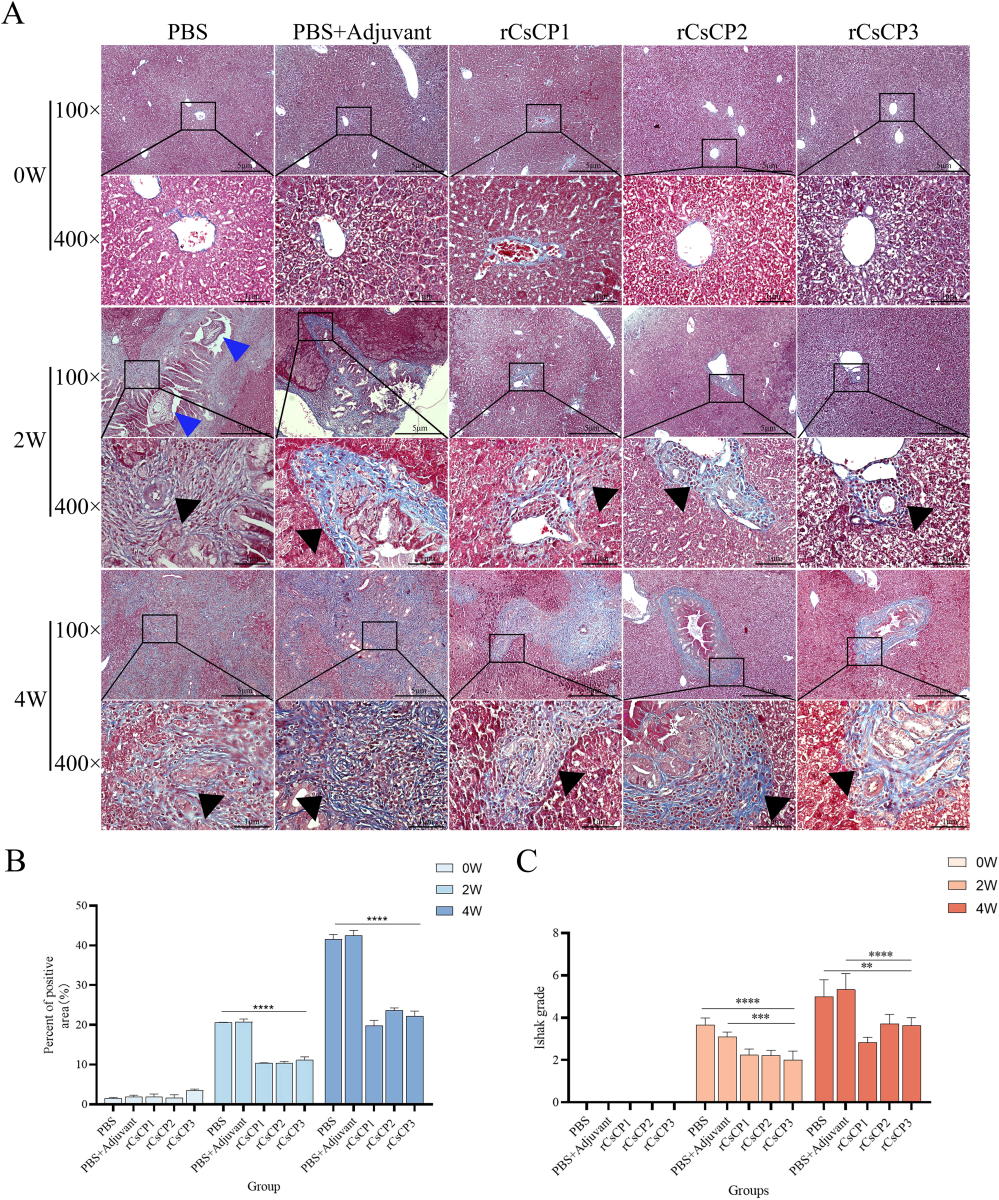
rCsCP1-3 immunization ameliorates fibrosis induced by *Clonorchis sinensis* infection. Fixed liver tissues were assessed for collagen deposition using Masson's trichrome staining (blue indicates collagen deposition areas). Infection with *Clonorchis sinensis* resulted in significant collagen deposition in bile duct areas, which was alleviated by immunization with rCsCP1-3 proteins **(A)**. Hepatic fibrosis was quantified using ImageJ **(B)** and the Ishak scoring system **(C)**. Blue triangles indicate *C*. *sinensis* juvenile worms; black triangles indicate collagen deposition areas. **P < 0.01, ***P < 0.001, ****P < 0.0001; Scale bar: 100 µm.

Masson’s staining of liver tissues showed a reduction in collagen fiber proliferation in the bile duct areas of the rCsCP1-3 immunized group compared to the PBS and PBS plus Freund’s adjuvant groups (**Figure 6A**), accompanied by a decrease in fibrotic area (**Figure 6A**). ImageJ analysis and Ishak scoring indicated that the rCsCP1-3 immunized group had significantly lower scores than the PBS and PBS plus Freund’s adjuvant groups at both the second and fourth weeks post-infection (**Figures 6B, C**). These results consistently demonstrated a lower degree of liver fibrosis in the rCsCP1-3 immunized group.

**Figure 7:**
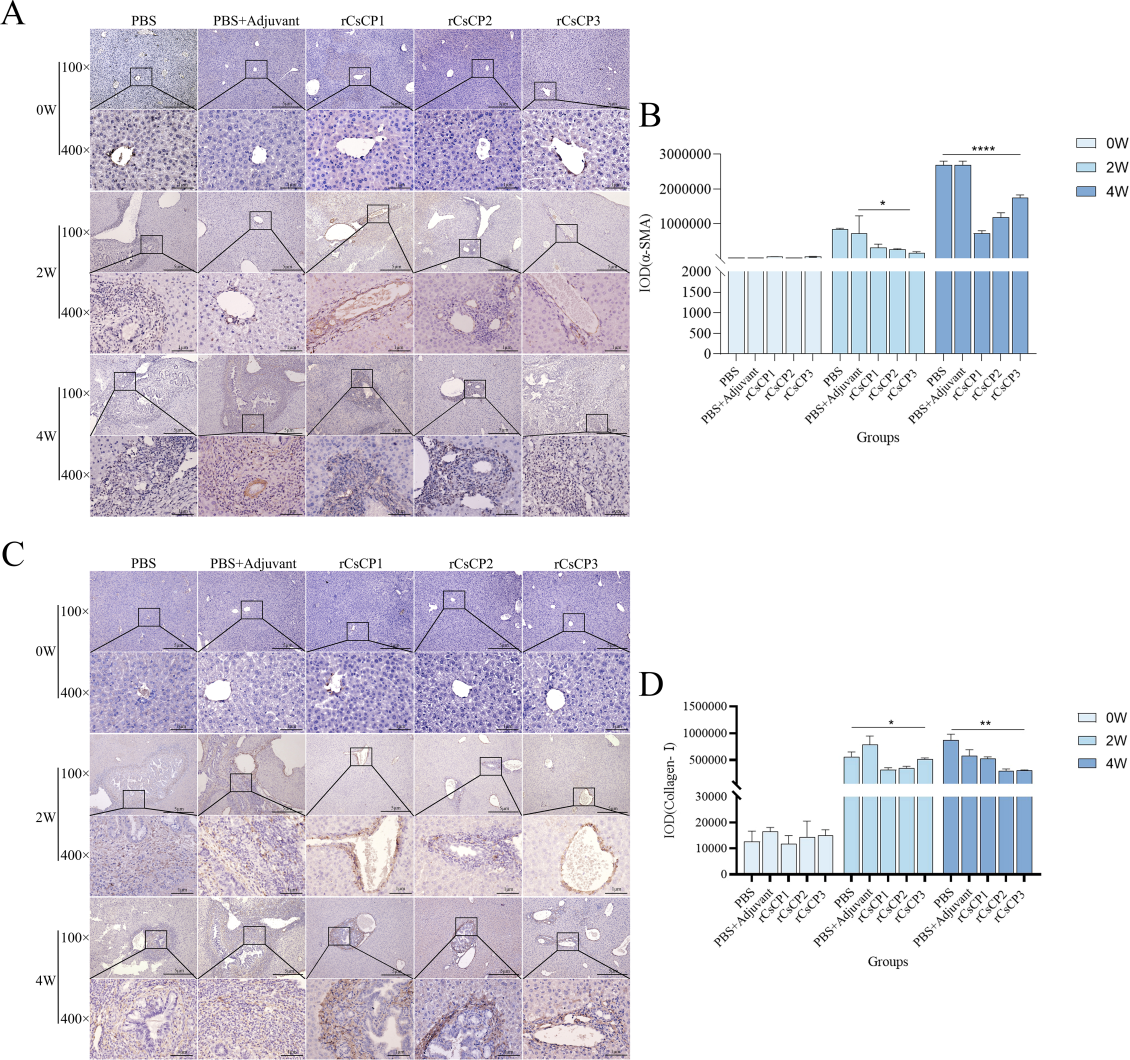
rCsCP1-3 immunization reduces the expression of α-SMA and anti-Collagen-I in *Clonorchis sinensis-*infected mouse liver tissues. Fixed liver tissues were stained with anti-α-SMA **(A)** and anti-Collagen-I **(C)** antibodies. The positive staining areas were quantified using ImageJ **(B, D)**. Following *Clonorchis sinensis* infection, α-SMA and Collagen-I staining intensity increased in the PBS and PBS plus Freund's adjuvant groups. In contrast, the rCsCP1-3 immunized group exhibited reduced staining intensity. The brown patr represents positive areas; *P < 0.05, **P < 0.01, ***P < 0.001, ****P < 0.0001. Scale bar: 100 µm.

Immunohistochemical analysis showed that the expression of α-SMA and Collagen I in the rCsCP1-3 immunized group was lower than in the PBS and PBS plus Freund’s adjuvant groups (**Figures 7A, C**). Statistical analysis of the positive staining areas confirmed that α-SMA and Collagen I expression was significantly reduced in the rCsCP1-3 immunized group compared to the PBS and PBS plus Freund’s adjuvant groups (*P* < 0.05) (**Figures 7B, D**). These results suggest that rCsCP1-3 immunization alleviates biliary damage and fibrosis in mice following *C. sinensis* infection.

### 3.6 Anti-rCsCP1-3 IgG antibody titer assay

**Figure 8:**
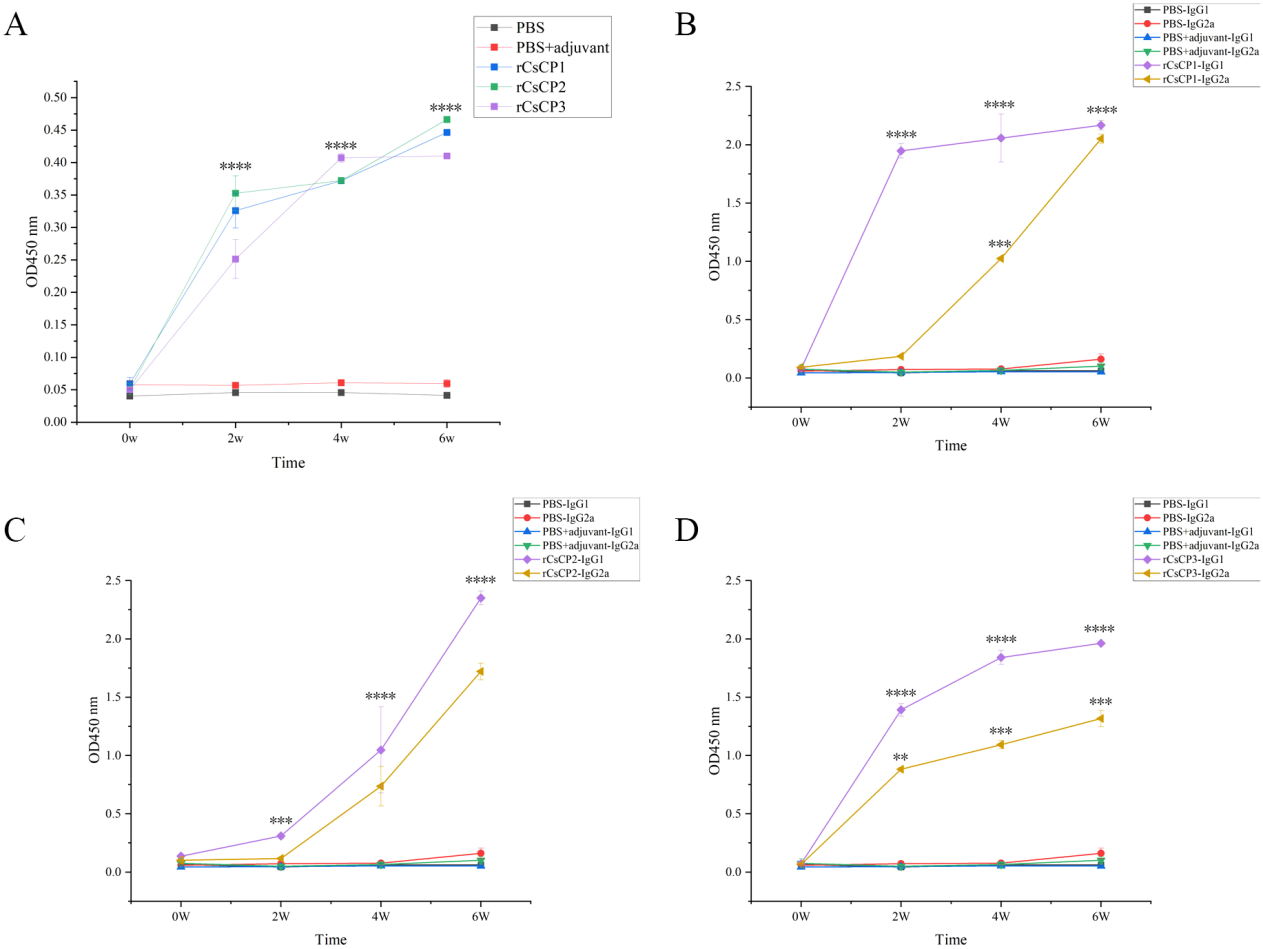
Specific IgG antibody levels induced by rCsCP1-3 immunization. Immunization with rCsCP1-3 rapidly induced specific IgG production **(A)**, including subclasses IgG1 **(B)** and IgG2a **(C, D)**. **P < 0.01, ***P < 0.001, ****P < 0.0001.

Antibody titer assays demonstrated that immunization with rCsCP1-3 induced a rapid production of specific IgG within two weeks post-infection, whereas no increase was observed in control groups receiving adjuvant or PBS (**Figure 8A**). Analysis of IgG subclasses, IgG1 and IgG2a, revealed a rapid rise in IgG1 levels in the rCsCP1 immunized group, with a deceleration in the second week. IgG2a exhibited a gradual increase in the first two weeks, followed by a sharp rise (**Figure 8B**). In the rCsCP2 immunized group, both IgG1 and IgG2a titers showed a steady increase in the first two weeks, with a rapid escalation thereafter (**Figure 8C**). In contrast, the rCsCP3 immunized group displayed an initial rapid increase in both IgG1 and IgG2a titers, followed by a decelerated rate of increase in the second week (**Figure 8D**).

### 3.7 Measurement of cytokine levels

**Figure 9:**
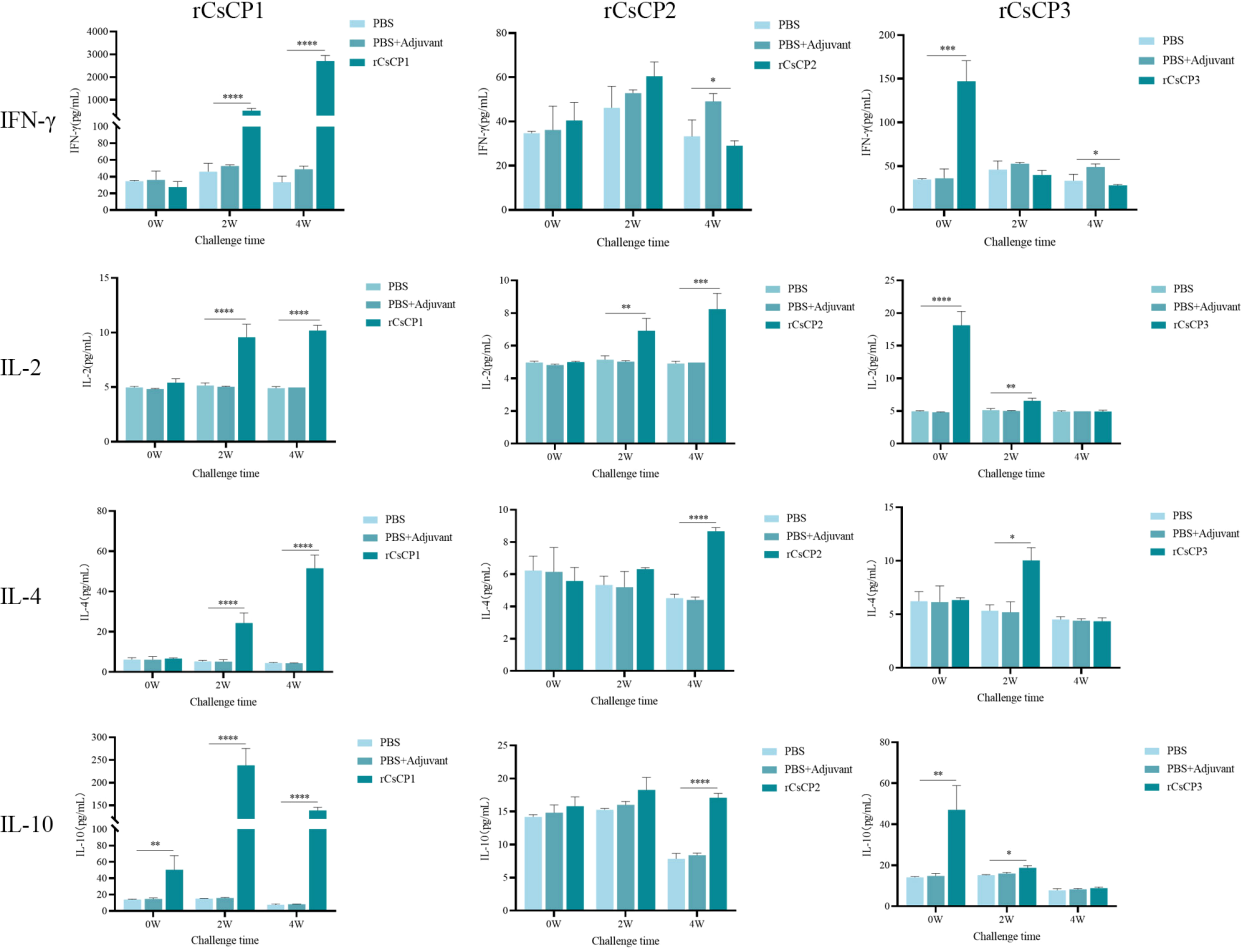
Cytokine secretion levels of IFN-γ, IL-2, IL-4, and IL-10. Compared to the PBS and PBS plus Freund's adjuvant control groups, the rCsCP1-immunized group showed a sharp increase in IL-10, IFN-γ, IL-2, and IL-4 levels within two weeks. The rCsCP2-immunized group induced a gradual increase in IFN-γ, IL-4, and IL-10 levels within four weeks. The rCsCP3-immunized group showed an initial increase in IFN-γ, IL-2, IL-4, and IL-10 levels within two weeks, followed by a rapid decrease, with no significant differences compared to the control groups. **P < 0.01, ***P < 0.001, ****P < 0.0001.

ELISA results indicated that in the rCsCP1 immunized group, IL-10 levels surged over 10-fold (11.73 ± 1.97)by the first week post-infection, compared to control groups, and remained elevated at the second week. Concurrently, IFN-γ, IL-2, and IL-4 increased significantly starting from the second week (*P* < 0.001), with IFN-γ expression being more than 80-fold(82.93 ± 12.86) higher than in control groups (**Figure 9**). In the rCsCP2 immunized group, IL-2 and IL-4 levels rose from the second and fourth weeks post-infection, while IFN-γ levels decreased noticeably, and IL-10 expression remained unchanged compared to controls (**Figure 9**). In the rCsCP3 immunized group, IFN-γ, IL-2, and IL-10 were elevated from the first week post-infection (P < 0.01), with IL-4 levels increasing in the second week. By the fourth week, no significant differences in the expression of IFN-γ, IL-2, IL-4, or IL-10 were observed between the immunized and control groups (**Figure 9**).

## 4 Discussion

This study revealed three key characteristics of CsCP1-3. Both CsCP1 and CsCP2 exhibit the highest expression in the NEJ stage, with CsCP1 showing over 200 times higher expression than in the adult and metacercarial stages. This suggests that CsCP1 may play a critical role in key processes during the NEJ stage, such as excystation, intestinal invasion, and nutrient absorption. Further analysis demonstrated that CsCP1 is widely distributed on the eggshell, the surface of metacercariae, the outer tegument and oral sucker of NEJ, as well as the intestine, tegument, and cell layers of adult worms. The outer tegument proteins have been shown to interact directly with immune cells, antibodies, and cytokines (35), and several tegumental proteins have been identified as potential vaccine candidates in flukes (28, 36, 37).

To evaluate the immunoprotective effects of CsCP1-3 following *C. sinensis* challenge and two booster immunizations, liver damage was assessed using hematoxylin and eosin (HE) staining, Masson’s trichrome staining, and immunohistochemical analysis of α-smooth muscle actin (α-SMA) and Collagen-I. Post-immunization results indicated that CsCP1-3 effectively reduced liver fibrosis progression and inflammatory cell infiltration. Compared to the CsCP1-3 immunized group, control mice displayed thicker bile ducts, more pronounced collagen fiber proliferation in the bile duct area, and higher levels of α-SMA and Collagen-I. These findings suggest that immunization with CsCP1-3 significantly mitigated liver tissue damage and reduced liver fibrosis induced by *C. sinensis* infection.

To preliminarily investigate the immunoprotective mechanisms of CsCP1-3, antibody and cytokine responses were evaluated following immunization. Immunization with CsCP1-3 induced a rapid production of IgG, with IgG subclasses IgG1 and IgG2a showing a gradual increase from 1 to 4 weeks post-infection. Previous studies on potential vaccine candidates for *C. sinensis* have linked protective immunity and worm reduction to elevated levels of IgG1/IgG2a (36, 38). Cytokine analysis revealed that the levels of IFN-γ, IL-2, IL-4, and IL-10 were significantly higher in the CsCP1-immunized group compared to the control group, particularly IFN-γ and IL-10, which were more than 10 times higher than in controls. IL-10, an important anti-inflammatory cytokine, plays a pivotal role in fibrosis and is primarily secreted by activated macrophages and dendritic cells, exerting inhibitory effects on fibrosis (39, 40). In *C. sinensis*-infected mice, serum cytokines IL-4 and IL-10 increased, with IL-10 showing a marked rise (41), while IFN-γ and IL-2 levels decreased. During liver fibrosis, Polarized macrophages release anti-inflammatory factor IL-10, influencing fibrosis progression (42). Moreover, IL-10 gene therapy has been shown to reverse thioacetamide-induced liver fibrosis in mice (43). The increased levels of IgG1/IgG2a antibodies and cytokines IFN-γ, IL-2, IL-4, and IL-10 following CsCP1 immunization suggest that CsCP1 elicits a mixed type 1/type 2 immune response, mitigating liver damage caused by *C. sinensis* infection. In previous studies, vaccine candidates for *C. sinensis* have achieved significant worm reduction and alleviated liver damage by inducing a mixed type 1/type 2 immune response (36, 44, 45).

In the CsCP2-immunized group, IgG1/IgG2a levels gradually increased, and IL-2 and IL-4 expression significantly surpassed that of the control group at four weeks post-infection. This suggests that CsCP2 also triggers a mixed type 1/type 2 immune response in the adult stage, reducing liver damage. In the CsCP3-immunized group, expression levels of IFN-γ, IL-2, IL-4, and IL-10 rose rapidly post-infection but subsequently dropped to levels not significantly different from the control by the second or fourth week. This suggests that the observed reduction in liver damage is likely attributed to a robust innate immune response, which is critical in triggering the subsequent adaptive immune response (46, 47).

## 5 Conclusion

This study characterized the expression and localization of three cysteine proteases (CsCP1-3) from *C. sinensis* across various life cycle stages of the parasite. Immunization with CsCP1-3 effectively reduced liver damage caused by *C. sinensis* infection, although the protective mechanisms varied among the three proteins. Notably, CsCP1 elicited a robust mixed type 1/type 2 immune response, highlighting its potential as a vaccine candidate. Future research will focus on detailed vaccine efficacy, including worm burden reduction rates and further exploration of the underlying protective mechanisms.

## Data Availability

The datasets presented in this study can be found in online repositories. The names of the repository/repositories and accession number(s) can be found in the article/**Supplementary Material**.
