# Correction: Characteristics and immunoprotective functions of three cysteine proteases from *Clonorchis sinensis*

**DOI:** 10.3389/fimmu.2025.1663826

**Published:** 2025-09-15

**Authors:** Yunliang Shi, Xiaoqin Li, Kai Liang, Ting Lu, Yu Chen, Yashi Lai, Yaoting Li, Shuai Wei, Shanshan He, Lili Tang, Dengyu Liu, Yanwen Li

**Affiliations:** ^1^ Parasitology Department, School of Basic Medical Sciences, Guangxi Medical University, Nanning, China; ^2^ Key Laboratory of Basic Research on Regional Diseases (Guangxi Medical University), Education Department of Guangxi Zhuang Autonomous Region, Nanning, China; ^3^ Department of Medical Laboratory, Shenzhen Longgang District Eighth People’s Hospital, Shenzhen, China; ^4^ Gastroenterology Department, Guangxi Zhuang Autonomous Region People’s Hospital, Nanning, China; ^5^ Department of Schistosomiasis Prevention and Control, Disease Prevention and Control Center of Hengzhou City, Hengzhou, China; ^6^ Department of Medical Laboratory, Hechi People’s Hospital, Hechi, China

**Keywords:** *Clonorchis sinensis*, cysteine proteases, characteristics, liver damage, immune protection

In the published article, there was an error in **Figure 4** as published. During the process of arranging the figures, the electron microscopy images and fluorescence photographs of the adult worm of anti-CSCP2 and anti-CSCP3 were placed in the wrong positions. They should be swapped. The corrected **Figure 4** and its caption: “Tissue localization of rCsCP1-3 at various developmental stages of Clonorchis sinensis” appear below.

**Figure 4:**
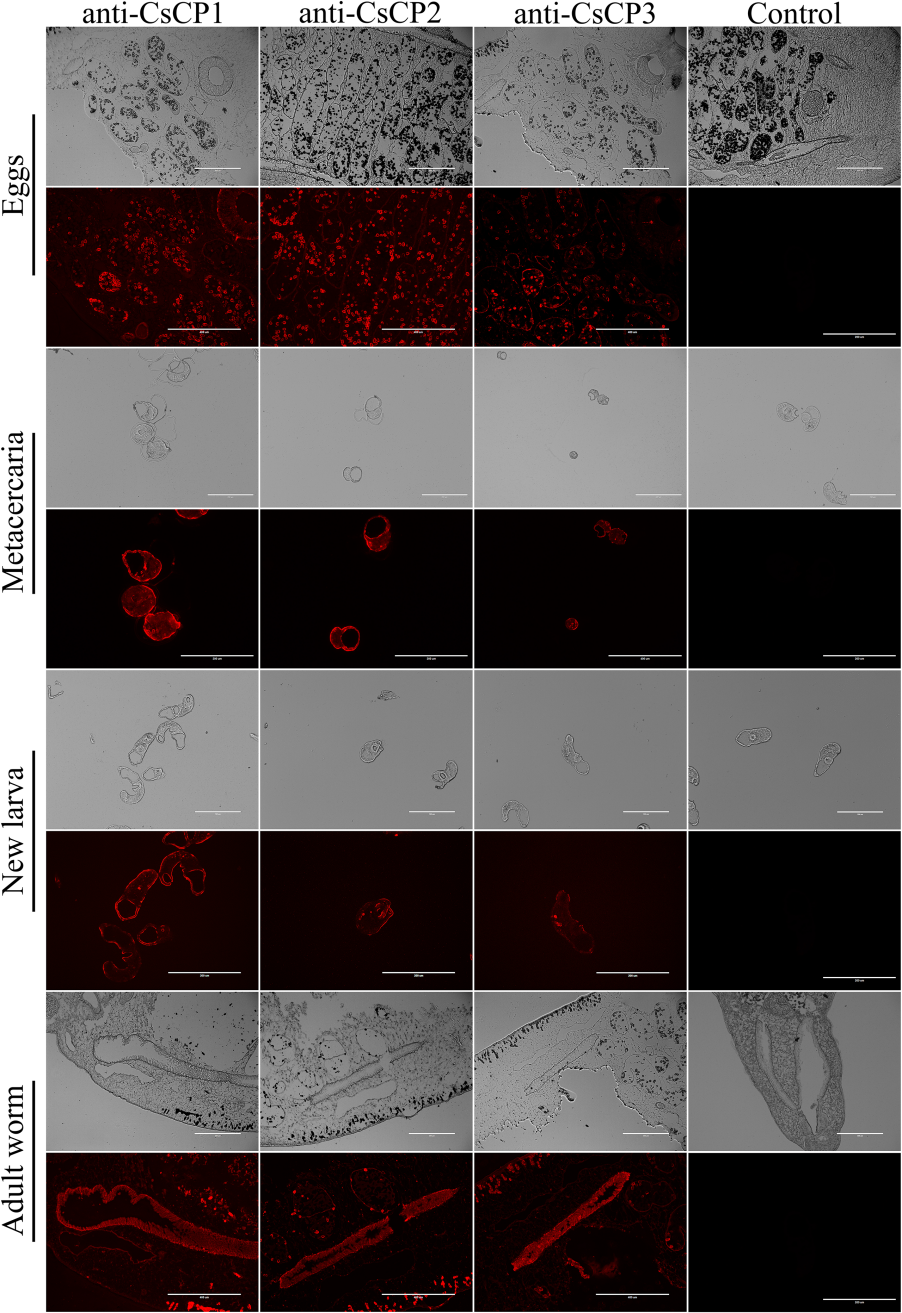
Tissue localization of rCsCP1-3 at various developmental stages of *Clonorchis sinensis*. Immunofluorescence localization of rCsCP1-3 distribution in eggs, metacercariae, newly excysted juveniles, and adult stages of *Clonorchis sinensis*.

The original version of this article has been updated.

