# Supplementary material for: Characteristics and immunoprotective functions of three cysteine proteases from Clonorchis sinensis
Source: Front Immunol. 2025 Apr 3;16:1550775. doi: 10.3389/fimmu.2025.1550775 (PMC12003271; doi:10.3389/fimmu.2025.1550775)
Supplement: Supplementary file 1 [file DataSheet1.pdf]

Linear epitope analysis of CsCP1-3 B cells

The results showed that CsCP1-3 predicted multiple linear B cell epitopes in both the cathepsin precursor peptide inhibition domain and the papain family domain (Supplementary Figure 1 and Table 2). Among them, the first 120 amino acid sequences of CsCP1-3 contain 4 linear B cell epitopes, and this sequence highly overlaps with the inhibitory domain of cathepsin precursor peptide (Supplementary Figure 1).

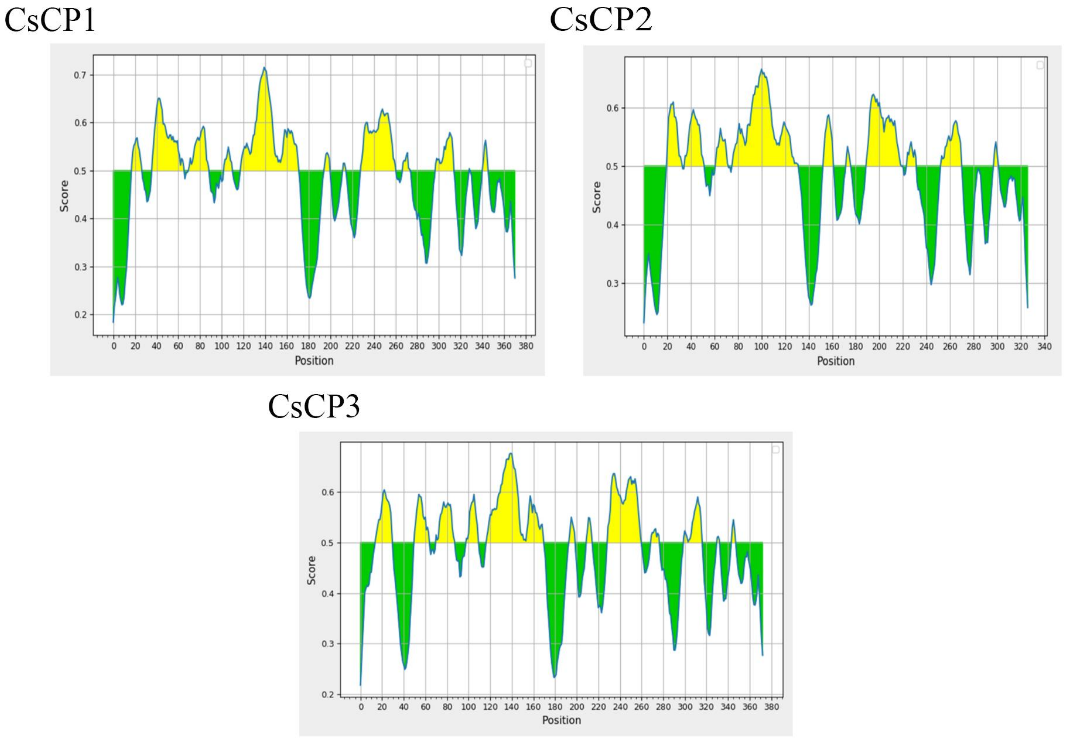

Supplementary Figure 1 B-cell linear epitope prediction of *Cs*CP1-3

Supplementary Table 2 Possible B-cell epitopes of CsCP1-3.

Number	*Cs*CP1	*Cs*CP2	*Cs*CP3
1	aa21-31	aa21-33	aa15-30
2	aa37-54	aa36-52	aa51-64
3	aa60-60	aa62-72	aa71-88
4	aa63-73	aa76-132	aa99-110
5	aa78-133	aa154-162	aa118-170
6	aa155-163	aa173-176	aa194-199
7	aa174-178	aa190-219	aa210-215
8	aa191-219	aa221-221	aa230-260
9	aa226-226	aa225-232	aa270-277
10	aa228-230	aa253-270	aa300-317
11	aa232-233	aa298-302	aa331-333
12	aa254-268		aa345-348
13	aa297-301		
