# Supplementary material for: Characteristics and immunoprotective functions of three cysteine proteases from Clonorchis sinensis
Source: Front Immunol. 2025 Apr 3;16:1550775. doi: 10.3389/fimmu.2025.1550775 (PMC12003271; doi:10.3389/fimmu.2025.1550775)
Supplement: Supplementary file 2 [file Table1.docx]

Primers for CsCP1-3 real time PCR

| Names | Primer sequences | Length（bp） |
| --- | --- | --- |
| CsCP1-F | 5’-GCGGATCCCAAGTTGAGCCTGACA-3’ | 164 |
| CsCP1-R | 5’-GGCTCGAGCTAAAACTTCTCGTTATCCATCGT-3’ |  |
| CsCP2-F | 5’-TTGCAGCTGGACTCCGACTA-3’ | 150 |
| CsCP2-R | 5’-TAAGAGGCCCGGTTTCCTTG-3’ |  |
| CsCP3-F | 5’-GCTTCAAGAGACGGCTCGAA-3’ | 151 |
| CsCP3-R | 5’-ATTGCACCAGTAGCTGAGAACG-3’ |  |
| β-Actin-F | 5’-ACCGTGAGAAGATGACGCAGA-3’ | 151 |
| β-Actin-R | 5’-ATCGGGACAGTATGGGTCACA-3’ |  |
